# Factors Affecting Occupational Balance Among Iranian Caregivers of Children With Cerebral Palsy: A Cross‐Sectional Study

**DOI:** 10.1155/oti/1041707

**Published:** 2026-10-22

**Authors:** Mahnaz Hejazi-Shirmard, Hosna Mohammadi, Marzieh Pashmdarfard

**Affiliations:** ^1^ Department of Occupational Therapy School of Rehabilitation Shahid Beheshti University of Medical Sciences Tehran Iran sbmu.ac.ir

**Keywords:** caregiver, cerebral palsy, occupational balance, quality of life

## Abstract

**Introduction:**

Children with cerebral palsy (CP) face lifelong motor, functional, and participation challenges, requiring continuous support for daily activities. Although caregiving can enhance family bonds, it often negatively affects caregivers′ physical and social well‐being, family dynamics, independence, and financial stability. The aim of the present study was to investigate the associations between occupational balance, caregiver burden, and related factors among parents of Iranian children with CP.

**Methods:**

This cross‐sectional study recruited 112 informal caregivers of children aged 2–18 years with CP from March to October 2023. Children′s functional status was assessed using classification systems for gross motor function (GMFCS), manual ability (MACS), communication function (CFCS), and cognitive status (SPARCLE). Caregivers′ burden, quality of life, and occupational balance were evaluated with the Caregiver Difficulties Scale (CDS), WHOQOL‐BREF, and OBQ‐11, respectively. Moreover, caregivers′ psychological well‐being was measured through the Perceived Stress Scale (PSS‐14) and Hospital Anxiety and Depression Scale (HADS). Data were analyzed using correlation analyses, independent *t*‐tests, one‐way ANOVAs, and hierarchical multiple linear regression model.

**Results:**

Caregivers′ occupational balance was significantly associated with key demographic and child‐related factors, including employment status, type of CP, and GMFCS level V. Psychological variables—anxiety/depression, perceived stress, caregiver burden, and quality of life—were also significant independent predictors. The final model explained 36.5% of the variance in occupational balance.

**Conclusion:**

Caregivers′ occupational balance is influenced by caregiver characteristics, psychological well‐being, and the severity of the child′s functional limitations. Therefore, comprehensive and multidisciplinary interventions targeting both parents and children—including rehabilitation, psychological support, and social services—are essential and urgently needed.

## 1. Introduction

Cerebral palsy (CP), the most common pediatric disability, describes a group of permanent disorders that affect the development of movement and posture, leading to long‐term activity limitations [1, 2]. Originating from nonprogressive disturbances in the developing fetal or infant brain [1, 2], CP is frequently associated with motor impairments as well as difficulties in sensation, perception, cognition, communication, behavior, and in some cases, epilepsy [3]. Children with CP often require lifelong and substantial assistance with daily activities, with the extent of needed support depending on the severity of their impairments and disabilities [2]. Essentially, CP is primarily managed within the home, where caregivers provide continuous support beginning at diagnosis and continuing into adulthood and beyond [4].

Although caregiving is a normal part of parenthood, caring for a child with a chronic health condition such as CP is often associated with negative health consequences for caregivers, including increased levels of depression, stress, anxiety, and reduced self‐efficacy [2]. Although caring for a child with CP can strengthen family bonds, it also negatively affects caregivers′ physical and social well‐being, family dynamics, independence, and financial stability [5]. Caregiver burden is the impact of caregiving on the primary caregiver, highlighting how responsibilities influence key aspects of their life, including social interactions, leisure, health, and personal privacy [6]. More specifically, it is a multidimensional reaction to the physical, psychological, emotional, social, and financial challenges tied to the caregiving role [7]. Caregiver burden has a profound impact on the physical and mental well‐being of caregivers, often leading to inadequate care and unmet patient needs [7]. These caregiving challenges are especially pronounced in developing countries, where limited access to basic services for individuals with disabilities exacerbates the challenges faced by caregivers [7]. Research has demonstrated that parents of children with disabilities, whether intellectual or physical, spend considerably more time on caregiving and have less leisure time than parents of typically developing children [8, 9]. On the other hand, emerging research indicates that maintaining occupational balance (OB) is particularly difficult for these parents because of the extensive demands of caregiving [10].

OB is a multidimensional concept that reflects a satisfying amount and variety of daily activities [10]. It involves maintaining a balance between activities performed with others and those done individually, between obligatory tasks and voluntary ones, and between energy‐restoring and energy‐consuming activities [10]. Dhas et al. explored OB among parents of children with disabilities and those with typically developing children. Their findings revealed statistically significant, though minimal, differences between the two groups, as well as a moderate association between OB and quality of life [10]. Research on OB in parenting is limited, and its associations with other factors, such as caregiver burden remain unclear. As understanding the associations between OB, caregiver burden, as well as the factors influencing them, is crucial for enhancing services for children with CP and their families, the aim of the present study was to investigate the associations between OB, caregiver burden, and other factors among parents of Iranian children with CP.

## 2. Materials and Methods

From March to October 2023, a total of 112 informal caregivers of children with CP were recruited for this cross‐sectional study. Inclusion criteria required that the child had a confirmed diagnosis of CP by a pediatric neurologist, was between 2 and 18 years of age, and that the caregiver was 18 years or older, capable of understanding instructions, and able to complete questionnaires. Caregivers were excluded if they had more than one child with a disability, cared for more than one disabled individual, or had severe acute or chronic physical or psychological conditions that significantly limited their ability to provide care. A priori sample size estimation was conducted using an expected medium effect size (*r* = 0.30), *α* = 0.05, and power = 0.80, indicating that a minimum of 84 participants was required [11]. The final sample of 112 exceeded this threshold.

Acknowledging the influence of caregivers′ socioeconomic and cultural backgrounds on perceived care burden and OB, we divided Tehran into five geographical regions (north, south, east, west, and central) to ensure representation of diverse living environments [12]. From each region, one to two rehabilitation centers or clinics were selected using convenience sampling based on accessibility and willingness to participate. This study received ethical approval from the Ethics Committee at the Shahid Beheshti University of Medical Sciences (IR.SBMU.RETECH.REC.1403.306), and written informed consent was obtained from all parents before the study started.

Demographic information for caregivers and children was collected using a structured questionnaire. Standardized instruments were also administered to assess caregiver‐ and child‐related outcomes; detailed descriptions of these measures are provided in the following subsections. All questionnaires were completed through face‐to‐face interviews conducted by an occupational therapist to ensure accuracy and consistency in data collection.

All assessments were conducted in the rehabilitation centers from which participants were recruited. Caregivers and children were evaluated during a single scheduled visit. A trained occupational therapist conducted face‐to‐face interviews with caregivers to complete the sociodemographic questionnaire and caregiver‐related measures. The functional status of the children was assessed by an occupational therapist experienced in using these classification systems. All evaluations were performed in a quiet room within the rehabilitation center to ensure comfort, privacy, and consistency across participants.

### 2.1. Sociodemographic Variables

Sociodemographic data were obtained for both caregivers and children. Caregiver variables included age, gender, marital status, education level, employment status, and number of children. Child‐related variables included age, gender, and type of CP. All sociodemographic variables were categorized according to standard demographic classifications for statistical analysis.

### 2.2. Caregiver′s Outcomes

Caregiver Difficulties Scale (CDS) is an assessment tool used to evaluate the caregiving burden experienced by informal caregivers. It comprises 25 items organized into four main domains of concerns for the child, impacts on the caregiver, support received for caregiving, and socioeconomic strains [7]. Each item is rated on a Likert scale from 0 to 4, with higher scores indicating a greater burden on the caregiver [13].

Occupational Balance Questionnaire‐11 (OBQ‐11) is a self‐report instrument designed to measure perceived OB. Each item is scored on a four‐point scale from “strongly disagree” (0 points) to “strongly agree” (3 points), resulting in a total score between 0 and 33. Higher scores indicate better OB [14, 15].

The World Health Organization Quality of Life‐BREF (WHOQOL‐BREF) is a brief instrument developed by the World Health Organization to evaluate quality of life. It includes 26 questions in four domains of physical health, psychological health, social relationships, and environmental health. Scores for each domain are transformed to a scale of 4–20, with higher scores indicating a better quality of life [16, 17].

Perceived Stress Scale (PSS) is one of the most widely used scales for evaluating psychological stress. The original version (PSS‐14) contains 14 items, with seven positive and seven negative statements, which are rated by participants on a 5‐point Likert scale. The scale assesses how individuals perceive their lives in terms of unpredictability, uncontrollability, and feelings of being overloaded during the previous month [18, 19].

Hospital Anxiety and Depression Scale (HADS) is a brief and widely used screening tool for psychological distress, particularly anxiety and depression, across diverse populations [20]. It consists of 14 questions in two subscales: anxiety and depression. Each subscale includes seven questions, rated from 0 to 3, with higher scores indicating greater levels of anxiety or depression [21].

### 2.3. Children′s Outcomes

Gross Motor Function Classification System (GMFCS) is a widely used classification system to describe the functional performance and gross motor function of children with CP. In this system, children are categorized into one of five levels, from I (most able) to V (most limited) [22].

Manual Ability Classification System (MACS) is a system used to classify children with CP based on their ability to use their hands to handle objects in daily activities. The MACS focuses on the child’s typical manual performance rather than their capacity. It categorizes children into five levels, ranging from Level I (handles objects easily) to Level V (requires total assistance) [23, 24].

Communication Function Classification System (CFCS) evaluates a child′s communication performance across five levels of everyday communication effectiveness, ranging from Level 1 (communicates effectively and efficiently) to Level 5 (rarely communicates effectively) [25, 26].

The SPARCLE was used to estimate the cognitive abilities (IQ) of children. Based on the SPARCLE project, IQ levels are defined according to the ICD‐10 classification. According to the parents′ responses to six questions about their child′s learning and play quality, the child′s IQ was categorized into one of three levels: less than 50, between 50 and 70, or greater than 70 [27].

### 2.4. Data Analysis

Statistical analyses were performed in SPSS Version 24.0 (SPSS Inc., Chicago, Illinois, United States). Quantitative variables (including Likert‐scale scores) were summarized using means and standard deviations (SD) because their distributions were evaluated for normality using the Shapiro–Wilk test. Variables that met normality assumptions were analyzed using parametric tests, including Pearson correlation coefficients, independent *t*‐tests, and one‐way ANOVAs for group comparisons. These tests were selected because they are appropriate for normally distributed continuous data and allow for the examination of linear associations and mean differences between groups. For variables that did not meet normality assumptions, nonparametric alternatives were applied, including Spearman correlation coefficients and nonparametric group comparison tests when appropriate. Categorical variables were summarized using frequencies and percentages.

Given the modest sample size and the number of categorical predictors, model stability was addressed using a theory‐driven variable selection approach. Only variables with theoretical justification and evidence of association in preliminary analyses were included in the regression model to avoid overfitting. Multicollinearity was assessed using tolerance and variance inflation factor (VIF) values, with no violations detected. Model assumptions were evaluated through inspection of residual distributions, standardized residuals, and the Durbin–Watson statistic, all of which indicated that the assumptions of normality, homoscedasticity, and independence of errors were met.

Hierarchical multiple linear regression analysis was conducted to examine the associations between OB and psychological distress (HADS), caregiver burden (CDS), perceived stress (PSS‐14), and quality of life (WHOQOL‐BREF), while controlling for demographic and child‐related characteristics. Categorical predictors with more than two groups were dummy‐coded prior to entry.

Model fit across blocks was evaluated using the coefficient of determination (*R* ^2^), adjusted *R* ^2^, change in *R* ^2^ (*Δ* *R* ^2^), overall F‐tests with degrees of freedom (df_1_, df_2_), and F‐change statistics. Unstandardized regression coefficients (B), standard errors (SE), 95% confidence intervals (95% CI), and *p* values were calculated for each predictor. Statistical significance was set at *p* < 0.05.

## 3. Results

### 3.1. The Characteristics of Children With CP and Their Caregivers

A total of 112 caregivers participated in the study. The mean age of caregivers was 41.27 ± 6.19 years, and the mean age of their children was 9.09 ± 3.13 years. Most caregivers were female (70.5%), whereas 29.5% were male. The majority of caregivers reported being married (96.4%), with only a small proportion identifying as divorced or widowed (3.6%). Additional demographic characteristics of caregivers and children are presented in Table 1.

**Table 1:** Demographic characteristics of the caregivers and children with CP and bivariate group comparisons of occupational balance (*n* = 112).

Characteristics	(*M* *e* *a* *n* ± *S* *D*) or *n*(%)	OB (*m* *e* *a* *n* ± *S* *D*) or r	*p*
Age of parents (years)	41.27 ± 6.18	*r* = –0.18	**0.04**
Age of children (years)	9.09 ± 3.13	*r* = 0.04	0.67
Parent gender			0.56
Male	33 (29.5)	17.61 ± 2.89	
Female	79 (70.5)	16.27 ± 2.76	
Child gender			—
Male	59 (52.7)	—	—
Female	53 (47.3)	—	—
Cognition level of child			—
≤ 50	34 (30.4)	—	—
50 < 70	43 (38.4)	—	—
≥ 70	35 (31.3)	—	—
Marital status			**0.03**
Divorced or widowed	4 (3.6)	16.28 ± 2.61	
Married	108 (96.4)	18.73 ± 2.06	
Education level of parents			0.74
Primary/Secondary	8 (7.1)	16.31 ± 2.52	
High school	49 (43.8)	17.13 ± 3.11	
University	37 (33.0)	18.01 ± 2.66	
Employment			**0.01**
Unemployed	64 (57.1)	15.35 ± 2.73	
Retired	2 (1.8)	17.53 ± 1.48	
Part time	6 (5.4)	17.19 ± 1.34	
Full time	40 (35.7)	18.58 ± 2.21	
Number of children			**0.04**
One	36 (32.1)	18.97 ± 3.21	
Two	50 (44.6)	17.84 ± 2.46	
Three	22 (19.6)	17.36 ± 2.75	
Four	4 (3.6)	17.75 ± 2.63	
Parent disease			**0.003**
Yes	15 (13.4)	17.40 ± 2.77	
No	97 (86.6)	16.36 ± 2.83	
Type of parent disease			—
Hypertension	2 (1.8)	—	—
Diabetes	7 (6.3)	—	—
Arthritis and osteoarthritis	2 (1.8)	—	—
Migraine	1 (0.9)	—	—
Spinal stenosis	1 (0.9)	—	—
Asthma	2 (1.8)	—	—
Type of CP			**0.05**
Spastic hemiplegia	21 (18.8)	18.81 ± 2.20	
Spastic diplegia	28 (25)	17.04 ± 3.02	
Spastic quadriplegia	52 (46.4)	15 ± 1.41	
Ataxia	9 (8)	17.67 ± 2.06	
Athetoid	2 (1.8)	17 ± 2.91	
GMFCS level of children with CP			**0.02**
I	6 (5.4)	19.83 ± 2.56	
II	52 (46.4)	17.37 ± 2.65	
III	25 (22.3)	17.68 ± 2.32	
IV	20 (17.9)	17.56 ± 4.12	
V	9 (8.0)	16.15 ± 2.81	
MACS level of children with CP			0.13
I	27 (24.1)	17.15 ± 2.93	
II	38 (33.9)	18.18 ± 2.44	
III	24 (21.4)	17.04 ± 2.74	
IV	14 (12.5)	16.29 ± 3.40	
V	9 (8.0)	17.11 ± 2.80	
CFCS level of children with CP			0.77
I	49 (43.8)	18.10 ± 2.42	
II	16 (14.3)	18 ± 3.03	
III	13 (11.6)	16.92 ± 2.56	
IV	26 (23.2)	16 ± 3.18	
V	8 (7.1)	16.75 ± 2.31	

*Note:* Parent disease type and child cognitive level were collected as demographic variables but were not included in the bivariate analysis; therefore, no OB means or *p* values are reported for these categories. Higher OB scores indicate better occupational balance. Bold values indicate statistically significant results.

Abbreviations: CFCS, Communication Function Classification System; CP, cerebral palsy; GMFCS, Gross Motor Function Classification System; MACS, Manual Ability Classification System; OB, occupational balance; r, Pearson correlation coefficient; SD, standard deviation.

### 3.2. The Descriptive Statistics of the Variables Under Study and Their Correlations

Table 2 displays the means, SD, and correlations of the studied variables. It reveals that the OB subscales were negatively correlated with the CDS, PSS‐14, and HADS subscales (*p* < 0.001). Additionally, all of the correlations between the OB subscales as well as those between the WHOQOL subscales were positive, moderate to strong, and statistically significant (*p* < 0.001).

**Table 2:** The means and standard deviations of the variables under study and their correlations (*n* = 112).

	Mean	SD	1	2	3	4	5
1. PSS‐14	27.05	7.28	1				
2. HADS total	16.43	6.97	0.32	1			
3. WHOQOL	12.71	2.13	−0.67	−0.28	1		
4. CDS	55.10	13.93	−0.62	−0.27	−0.69	1	
5. Occupational balance	17.37	2.81	−0.63	−0.32	0.71	−0.69	1

*Note:* All correlations are significant at *p* < 0.001 (two‐tailed).

Abbreviations: CDS, caregiver difficulties scale; HADS, Hospital Anxiety and Depression Scale; OB, occupational balance; PSS‐14, Perceived Stress Scale‐14; SD, standard deviation; WHOQOL, World Health Organization′s Quality of Life Questionnaire.

### 3.3. The Relationship of OB With Demographic Characteristics

Table 1 also outlines the association between parents′ demographic characteristics and their OB, as measured through bivariate analyses. The Pearson correlation coefficients revealed a significant negative relationship between parents′ age and OB. University‐educated parents scored higher in OB compared with those with primary or secondary education. Similarly, employed parents had higher OB scores than unemployed parents. Additionally, parents of children with quadriplegic CP reported lower OB scores compared with those whose children had other forms of CP. Divorced or widowed parents, as well as parents of children at GMFCS Level V, also experienced lower OB. Child age was examined as a demographic variable; however, it did not show a significant association with OB in the preliminary analysis and therefore was not included in the regression model to avoid over‐adjustment.

### 3.4. Multiple Linear Regression Analysis

A two‐block hierarchical multiple linear regression was performed to evaluate predictors of OB among caregivers of children with CP. In Block 1, demographic and child‐related characteristics were entered. The initial model accounted for 10.0% of the variance in OB (*R* = 0.316, *R* ^2^ = 0.100, Adjusted *R* ^2^ = 0.038, F (7,104) = 1.652, *p* = 0.128). Within Block 1, caregiver employment (B = 4.400, *p* = 0.040), caring for a child with nonquadriplegic CP (*B* = 2.800, *p* = 0.002), and GMFCS Levels I–IV (*B* = 1.420, *p* = 0.020) significantly predicted higher OB.

In Block 2, psychological distress, caregiver burden, perceived stress, and quality of life were added. The addition of Block 2 accounted for a significant proportion of additional variance, *Δ* *R* ^2^ = 0.265, F − Change (4,100) = 10.433, *p* < 0.001. The overall model was statistically significant (*F* (11,100) = 5.223, *p* < 0.001), explaining 36.5% of the total variance in OB (*R* = 0.604, *R* ^2^ = 0.365, Adjusted *R* ^2^ = 0.295).

In the final model, HADS scores (*β* = −0.320, *p* < 0.001), CDS scores (*β* = −0.280, *p* < 0.001), and PSS‐14 scores (*β* = −0.270, *p* < 0.001) were significant negative predictors, whereas WHOQOL scores (*β* = 0.205, *p* < 0.001) significantly predicted higher OB. The significance of demographic variables was attenuated in Block 2, indicating that psychological burden and quality of life mediate the relationship between demographic characteristics and caregiver OB (Table 3).

**Table 3:** The results of the hierarchical multiple linear regressions for the factors related to the occupational balance of the parents of children with CP.

	Occupational balance			
	B (SE)	95% CI		*p*
		Lower bound	Upper bound	
Block 1: demographics				
Age	−0.27 (0.14)	−0.54	−0.00	0.06
Marital status (single = 0^a^)	−2.10 (2.33)	−6.61	2.41	0.35
Education level (nonacademic = 0^a^)	1.60 (2.05)	−2.42	5.65	0.40
Employment (unemployed = 0^a^)	4.40 (2.03)	0.48	8.32	**0.04**
Type of CP (quadriplegia = 0^a^)	2.80 (0.88)	1.05	4.55	**0.002**
GMFCS level of children with CP (level V = 0^a^)	1.42 (0.60)	0.23	2.61	**0.02**
Child number (four children = 0^a^)	2.50 (2.67)	−2.60	7.60	0.32
Model characteristics	*R* = 0.316, *R* ^2^ = 0.10, ${R^{2}}_{\text{adj}}=0.038$, *F* (7, 104) = 1.652, *p* = 0.128			
Block 2: PSS‐14, CDS, HADS, and WHOQOL				
HADS	−0.31 (0.08)	−0.47	−0.15	**< 0.001**
CDS	−0.26 (0.08)	−0.42	−0.10	**< 0.001**
PSS‐14	−0.25 (0.06)	−0.37	−0.13	**< 0.001**
WHOQOL	0.27 (0.07)	0.13	0.41	**< 0.001**
Model characteristics	*R* = 0.604, *R* ^2^ = 0.365, ${R^{2}}_{\text{adj}}=0.295$, *F* (11, 100) = 5.223, *p* < 0.001			
Change statistics	*Δ* *R* ^2^ = 0.265, F − change (4, 100) = 10.433, *p* < 0.001			

*Note:* Bold values indicate statistically significant results.

Abbreviations: B, unstandardized regression coefficient; CDS, caregiver difficulties scale; CI, confidence interval; CP, cerebral palsy; GMFCS, Gross Motor Function Classification System; HADS, Hospital Anxiety And Depression Scale; OB, occupational balance; PSS‐14, Perceived Stress Scale‐14; SE, standard error; WHOQOL, World Health Organization Quality of Life scale.

^a^Reference categories: single (marital status), nonacademic (education), unemployed (employment), spastic quadriplegia (CP type), GMFCS Level V, and four children (child number).

### 3.5. OB

In Block 1 of the hierarchical regression, higher age, being unemployed, having four children, having a child with quadriplegic CP, and having a child with GMFCS level V were significantly associated with lower OB scores (Table 1). In Block 1, the demographic variables explained 10% of the variance in OB (*R* ^2^ = 0.10), corresponding to a multiple correlation coefficient of *R* = 0.316.

In Block 2, psychological and quality‐of‐life variables were added to the model. OB scores showed negative associations with PSS‐14, CDS, and HADS scores, and a positive association with WHOQOL scores. The addition of these variables significantly improved the model (*R* ^2^ = 0.365, F − Change = 31.87, *p* < 0.001), indicating that they accounted for an additional 36.5% of the variance in OB.

The 95% confidence intervals for all regression coefficients are presented in Table 3. For all statistically significant predictors, the confidence intervals did not cross zero, supporting the stability and robust associations with OB.

## 4. Discussion

Children with CP require consistent special care and attention due to the challenges and limitations associated with their condition. Their extraordinary needs often place significant demands on parents, leading to physical and psychological strain. This burden can adversely impact parents′ lives, including their social, cultural, and professional activities [28], and ultimately can disrupt their OB. Investigating the relationships between OB and its influencing factors is vital for improving support services for children with CP and their caregivers. To the best of our knowledge, this study is the first to investigate OB and its related factors among Iranian caregivers of children with CP.

Our study revealed a significant negative correlation between OB in caregivers of children with CP and parents′ age, the number of children, and the child′s GMFCS level. On the other hand, there was a statistically significant correlation between OB and parents′ marital status and employment status.

Beyond the correlation findings, in Block 1 of the regression analysis, OB was significantly associated with caregiver employment, nonquadriplegic CP type, and lower child functional impairment. However, when psychological distress, perceived stress, caregiver burden, and quality of life were entered in Block 2, these demographic associations were attenuated. This indicates that the impact of child functional severity and caregiver background on OB is largely mediated through psychological distress, caregiving burden, and overall quality of life.

Günal et al. conducted a comparative study between mothers of children with CP and those of typically developing children, and found that mothers of children with CP experienced a reduced overall OB, as well as an imbalance between obligatory and voluntary activities. Their research also revealed significant differences in various aspects of quality of life between the two groups, including emotional responses, social isolation, and sleep patterns. Additionally, a correlation was identified between OB and the overall quality of life score [29]. In alignment with these findings, our study similarly demonstrated a positive association between OB and quality of life. Caregivers often provide near‐constant assistance for their children′s mobility, feeding, and toileting needs. In some cases, they are also required to accompany their children to school because of an insufficient number of caregivers within educational settings. Consequently, they frequently neglect their own physical, emotional, and social well‐being. Moreover, in prioritizing their caregiving responsibilities, they may inadvertently overlook the needs of other family members [4]. On the other hand, parents of children with CP may experience feelings of abandonment and discrimination, leading them to reduce or entirely withdraw from social interactions [4]. Subsequently, this imbalance in daily routines and meaningful occupations can negatively impact an individual′s quality of life by diminishing their overall sense of fulfillment and satisfaction.

We also found a significant negative association between OB and psychological factors, including anxiety, depression, perceived stress, and caregiving burden. Parents of children with CP are at an elevated risk of developing mental health conditions such as depression and anxiety [30]. These challenges may reduce their motivation to engage in meaningful activities, ultimately disrupting their OB.

The multiple regression analysis revealed that OB decreased with older parental age, unemployment, and greater parental psychological distress. As parents age, their OB seems to decrease, as caregiving becomes more demanding, parental capabilities diminish, and health complications increase. Albayrak′s study identified a significant positive correlation between parental age and the caregiving burden experienced by parents of children with CP [31]. However, Dhas et al. did not find a correlation between OB and parental age, which could be attributed to its small sample size [10]. Furthermore, our findings indicate that psychological well‐being—including stress, anxiety, and depression—plays a central role in shaping OB among caregivers. Parents experiencing higher psychological distress reported lower OB. Previous studies have demonstrated that parents′ overall health significantly influences caregiver burden [31, 32]. Accordingly, it can be inferred that health‐related challenges may complicate caregiving tasks, extend caregiving time, and ultimately contribute to a disruption in OB. Finally, it appears that university‐educated or employed parents may experience better OB, potentially due to increased access to resources, more effective coping strategies, and stronger social support networks. These factors can mitigate the demands of caregiving by enhancing resilience, facilitating engagement in meaningful activities, and reducing the overall burden associated with caregiving responsibilities.

The multiple regression analysis also showed that OB was associated with caring for a child with quadriplegic CP and having a child at GMFCS Level V. It has been suggested that children with more severe forms of CP frequently present with a greater number of comorbid conditions and are less likely to attain independence in ambulation, communication, and self‐feeding [30]. They are also less likely to be included in educational services and are therefore more dependent in daily activities and require more parental assistance [30]. Palisano et al. suggested that parents of children with severe motor impairments faced greater challenges in social and leisure activities, and social participation was more challenging among parents of children requiring significant physical assistance, particularly those with communication and learning difficulties [33]. The association between the severity of disability in children with CP and the OB of parents was not examined in the Dhas study; however, several studies have reported associations between gross motor functions, manual skills, communication abilities, functional independence, and the number of functional deficits in children with CP and the caregiver burden experienced by their parents [7, 28, 31, 32, 34]. These factors seem to similarly disrupt OB, particularly through increasing caregiver burden.

This study shows that parents′ OB is influenced not only by their own characteristics but also by the motor and functional challenges faced by their children with CP. These associations highlight the importance of comprehensive, transdisciplinary services that address both child needs and caregiver well‐being, ultimately supporting families in managing daily demands and promoting more balanced participation in everyday life. Although this study contributes to the limited body of research on the OB of parents caring for children with CP, several limitations should be acknowledged. The relatively small sample size and the uneven distribution of demographic variables—such as caregivers′ gender and marital status—restricted the ability to conduct subgroup analyses. Moreover, previously reported gender‐related differences in OB may have influenced the results [14]. Although children aged 2–18 years were included to represent the full pediatric age range, caregiving demands vary across developmental stages, and this wide age range may have affected the findings. The sample size was insufficient to examine age‐specific patterns, underscoring the need for future studies with larger cohorts to explore developmental differences in caregiving burden. Additionally, caregivers responsible for more than one child with a disability were excluded to avoid substantial confounding due to the markedly higher burden typically experienced by this group. However, this decision limits the generalizability of the results, as these families may face unique and more intense challenges. Their small number in the recruitment pool also prevented separate analysis. Future research should intentionally include and investigate this high‐burden subgroup. Finally, the use of convenience sampling reduces the generalizability of the findings. Random sampling was not feasible due to significant logistical and time constraints; nevertheless, future studies may improve the generalizability of findings by employing more rigorous sampling methods.

## 5. Conclusion

OB among caregivers of children with CP is significantly shaped by employment status, child functional severity (quadriplegia and GMFCS Level V), and psychological factors including stress, anxiety/depression, caregiver burden, and quality of life. These findings underscore the need for comprehensive, multidisciplinary interventions that simultaneously address child‐related functional needs and caregiver well‐being. Integrating rehabilitation services for the child with psychological, social, and occupational support for caregivers may help reduce caregiving burden, strengthen coping strategies, and promote more balanced participation in daily life for the entire family.

## Funding

No funding was received for this manuscript.

## Conflicts of Interest

The authors declare no conflicts of interest.

## Data Availability

The data that support the findings of this study are available from the corresponding author upon reasonable request.
